# Role of MAPK in apolipoprotein CIII-induced apoptosis in INS-1E cells

**DOI:** 10.1186/1476-511X-8-3

**Published:** 2009-02-05

**Authors:** E-ri M Sol, Tea Sundsten, Peter Bergsten

**Affiliations:** 1 Department of Medical Cell Biology, Uppsala University, Uppsala, Sweden

## Abstract

**Background:**

Individuals with type 2 diabetes mellitus (T2DM) have elevated levels of circulating apolipoprotein CIII (apoCIII). ApoCIII plays an important role for plasma triglyceride levels and elevated levels of the apolipoprotein have been connected with dyslipidemia in T2DM subjects. In addition, apoCIII has been linked to enhanced β-cell apoptosis. The present study was undertaken to investigate apoptotic mechanisms induced by the apolipoprotein.

**Results:**

ApoCIII (10 μg/ml) enhanced apoptosis 2-fold in insulin-producing INS-1E cells after 24 hours exposure to the apolipoprotein. At this time point phosphorylation of mitogen activated protein kinase (MAPK) p38 had doubled but ERK1/2 and JNK were not activated. Instead, ERK1/2 showed rapid and transient phosphorylation (2-fold after 0.5 hour). No JNK phosphorylation was observed. In support of a role of activation of not only p38 but also ERK1/2 in apoCIII-induced apoptosis, inclusion of p38 inhibitor SB203580 (10 μM) or ERK1/2 inhibitor PD98059 (100 μM) normalized apoptosis. Whereas influx of Ca^2+ ^was linked to apoCIII-induced ERK1/2 activation, pro-apoptotic protein CHOP/GADD of the unfolded protein response (UPR) was not affected by apoCIII.

**Conclusion:**

It is suggested that elevated circulating apoCIII levels may contribute to β-cell apoptosis via activation of p38 and ERK1/2 in individuals with T2DM. Therapies aiming at normalizing levels of apoCIII could be beneficial not only for the function of the β-cell but also for cardiovascular protection.

## Background

Type 2 diabetes mellitus (T2DM) is a disease involving both genetic and environmental factors [1,2] resulting in elevated circulating levels of glucose and lipids [3]. The negative effects of these elevated nutrient levels on β-cell function and mass are well documented [4-7]. Although causes of both genetic and environmental origin have been linked to these disturbances, the mechanisms remain to a great extent undefined, which is at least partly due to the complexity of T2DM. To dissect the multifactorial etiology of the disease studies have been undertaken, where analysis of changes in circulating levels of multiple proteins has been conducted by proteomic methodology [8-10]. In one of the studies the aim was to identify differentially displayed circulating proteins in individuals with T2DM potentially contributing to development of impaired β-cell function [9]. The experimental approach stems from work, where alterations in levels of circulating proteins, e.g. gut hormones and cytokines, have been demonstrated to have effects on β-cell function and mass [11,12]. To this aim serum samples from subjects with newly diagnosed diabetes with family history of diabetes (FHD) and impaired β-cell function, and from healthy individuals with no FHD and well functioning β-cells were protein profiled. Several circulating proteins were differentially displayed in the newly diagnosed T2DM individuals [9]. In the present study we have investigated mechanisms by which one of the identified proteins, apolipoprotein CIII (apoCIII), affects the insulin-producing cell. The choice of protein was based on the observed up-regulation of apoCIII in the circulation in individuals with T2DM [9,13,14] and enhanced apoptosis in an insulin-secreting cell line exposed to the apolipoprotein [15]. In the present study we hypothesized that apoCIII-induced β-cell apoptosis was connected to activation of mitogen-activated protein kinases (MAPKs) and/or induction of the pro-apoptotic protein CCAAT/enhancer-binding protein homologous protein/growth arrest and DNA-damage 153 (CHOP/GADD153) of the unfolded protein response (UPR), mechanisms which have been demonstrated to be activated in β-cell apoptosis [7,16-19]. The results indicate that MAPKs p38 and extracellular signal-regulated protein kinases 1 and 2 (ERK1/2), but not c-Jun NH_2_-terminal kinase (JNK) or CHOP/GADD153 play a role in apoCIII-induced β-cell apoptosis.

## Methods

### Chemicals and reagents

Reagents of analytical grade and MilliQ water were used. ApoCIII was purchased from Meridian Life Science (Saco, MA). PD98059, SB203580, verapamil, tolbutamide, thapsigargin and protease inhibitory cocktail (Sigma P-8340) were obtained from Sigma (St. Louis, MO). RPMI 1640 culture medium, fetal calf serum (FCS), sodium pyruvate, glutamine, penicillin and streptomycin were purchased from Invitrogen (Carlsbad, CA). Culture flasks and plates were from BD Biosciences Labware (Franklin Lakes, NJ).

### Cell culture

Rat insulinoma INS-1E cells, supplied by Dr. P Maechler, Geneva University, Switzerland [20], were cultured in a humidified atmosphere containing 5% CO_2 _in RPMI 1640 medium supplemented with 10 mM HEPES, 10% (v/v) heat-inactivated FCS, 2 mM glutamine, 100 U/ml penicillin, 100 μg/ml streptomycin, 1 mM sodium pyruvate and 50 μM β-mercaptoethanol. Sub-confluent INS-1E cells were exposed to 10 μg/ml apoCIII for 24 hours. In addition, PD98059 (100 μM) or SB203580 (10 μM) was included into the culture medium 0.5 hour prior to introducing apoCIII. Verapamil (100 μM) was added to INS-1E cells for 5 minutes and then withdrawn before exposing the cells to apoCIII. Tolbutamide (1 mM) was added into the medium and present throughout the culture period. As positive control for CHOP/GADD153, 300 nM thapsigargin was added to the culture medium and was present during the whole culture period. The positive control for the different MAPKs were obtained by exposing the cells for 10 minutes at 5 mW/cm^2 ^of UV-irridation (E_max _= 310 nM) with a transilluminator (San Gabriel, CA), followed by additional 10 minutes in the incubator before sample preparation.

### Protein measurements

Protein extracts were prepared by lysis of INS-1E cells with a buffer composed of 150 mM NaCl, 50 mM Trizmabase, 1% Triton X100, 0.25% Na-deoxycholate, 1 mM Na_3_VO_4_, 2 mM EGTA and a protease inhibitory cocktail. Extracts were subjected to SDS-PAGE, transferred to PVDF-membrane and immunoblotted. Antibodies against phosphorylated and un-phosphorylated p38, ERK1/2 and JNK were obtained from Cell Signaling (Beverly, MA). The CHOP/GADD153 and β-actin antibodies were purchased from Santa Cruz Biotechnology (Santa Cruz, CA). The immuno-reactive bands were visualized by chemiluminescence ('ECL Plus' or 'ECL Advance', GE Healthcare, Uppsala, Sweden) according to the manufacturer's protocol, imaged with Fluor-S MultiImager MAX (Bio-Rad, Hercules, CA). After imaging, the PVDF membranes were quantified with Quantity One software (BioRad). The expression level of each protein was normalized to un-phosphorylated MAPK and/or β-actin.

### Apoptosis measurements

DNA fragmentation of INS-1E cells was evaluated by determining oligonucleosome formation using the Cell Death Detection ELISA^PLUS ^kit (Roche Diagnostics, Mannheim, Germany). Measurements were related to DNA content and expressed as fold of optical density obtained for control cells cultured in the absence of apoCIII.

### Statistical analysis

Results are presented as means ± SEM (apoptosis) or representative blots (MAPKs and CHOP/GADD153) for four independent experiments. Western blots were densitometrically analyzed. Differences between groups are assessed by ANOVA followed by Tukey's post hoc test for apoptosis measurements and Student's t-test for immunoblots. A probability level of p < 0.05 was considered to be statistically significant.

## Results

### ApoCIII-induced apoptosis and MAPKs

**Figure 1:**
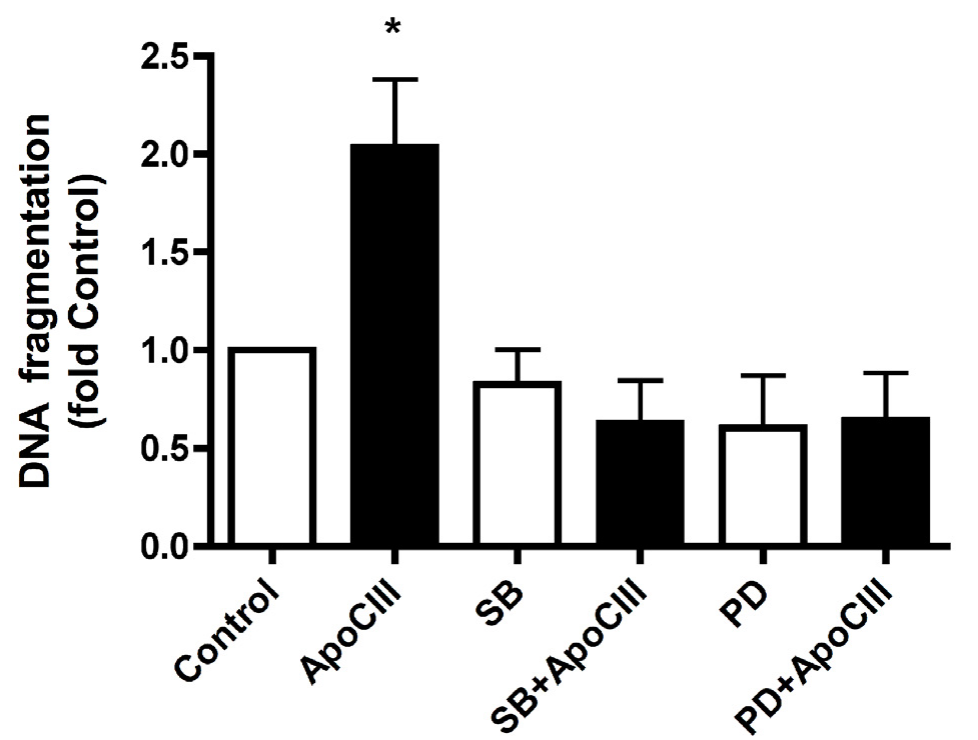
**ApoCIII-induced apoptosis**. INS-1E cells were cultured for 24 hours in the absence or presence of 10 μg/ml apoCIII. Cells were exposed to 10 μM of the p38 inhibitor SB203580 (SB) or 100 μM of the ERK1/2 inhibitor PD98059 (PD) during 0.5 hours prior to apoCIII treatment as indicated. After culture, apoptosis was measured as DNA fragmentation and normalized to DNA content. *P < 0.05 denotes effect of apoCIII.

**Figure 2:**
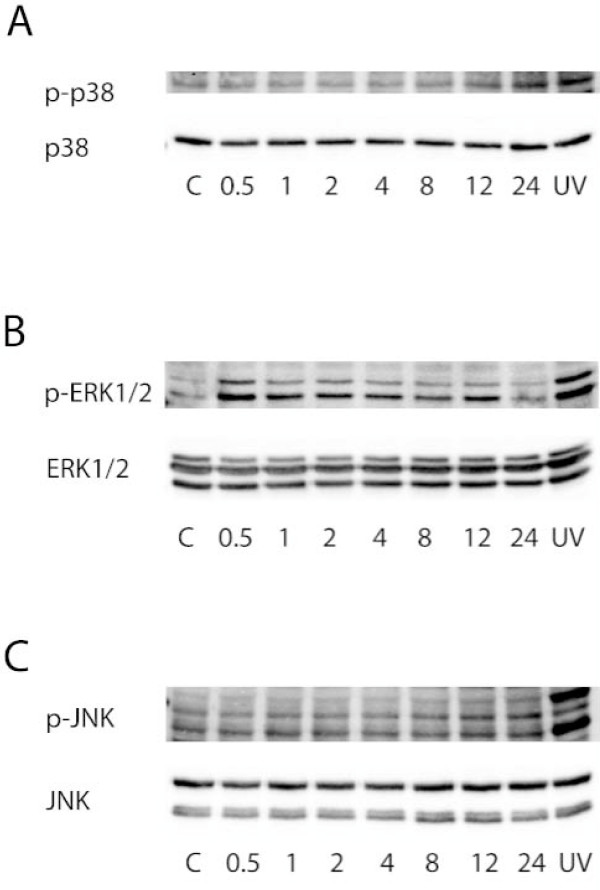
**ApoCIII-induced MAPK-activation**. Levels of phosphorylated p38 (A), ERK1/2 (B) and JNK (C) in apoCIII-treated INS-1E cells were measured at the indicated time points. Cells exposed to ultraviolet light (UV) were used as positive control.

Apoptosis was increased by 2-fold when INS-1E cells were exposed to apoCIII for 24 hours (Fig 1). To investigate underlying mechanisms of the apoCIII-induced rise in apoptosis, activation of MAPKs p38, ERK1/2 and JNK in INS-1E cells were measured at 0, 0.5, 1, 2, 4, 8, 12 and 24 hours after introduction of the apolipoprotein. Levels of p-p38 were low in the cells at onset of the apoCIII-exposure and were then progressively increasing for the duration of the experiment manifested as 2-fold rise (p < 0.05) after 24 hours (Fig 2A). To determine if activation of the MAPK was causually related to apoCIII-induced apoptosis, cells were exposed to the inhibitor of p38 phosphorylation SB203580 prior to introducing apoCIII. Administration of the inhibitor normalized p-p38 levels (not shown), which was accompanied by reduction of apoptosis to control levels in apoCIII-exposed cells (Fig 1). The inhibitor alone had no effect on apoptosis.

Involvement of ERK1/2 in apoCIII-induced apoptosis in INS-1E cells was also investigated. Levels of p-ERK1/2 were elevated 2-fold (p < 0.05) already after 0.5 hour and declined thereafter (Fig 2B). After 24 hours, when the apoCIII-induced rise in apoptosis was determined, ERK1/2 levels had returned to control levels. To investigate whether the early rise in p-ERK1/2 was contributing to apoCIII-induced apoptosis, cells were exposed to the inhibitor of ERK1/2 phosphorylation PD98059 prior to introducing apoCIII. Pre-exposing the cells to the inhibitor prevented the apoCIII-induced rise in p-ERK1/2 (not shown) and reversed apoptosis in the presence of the apolipoprotein (Fig 1). The inhibitor alone had no effect on apoptosis. Thirdly, phosphorylation of JNK was measured in INS-1E cells exposed to apoCIII. No activation of the MAPK by the apolipoprotein was observed during the 24-hour exposure time (Fig 2C).

### ApoCIII-induced ERK1/2 activation and calcium

**Figure 3:**
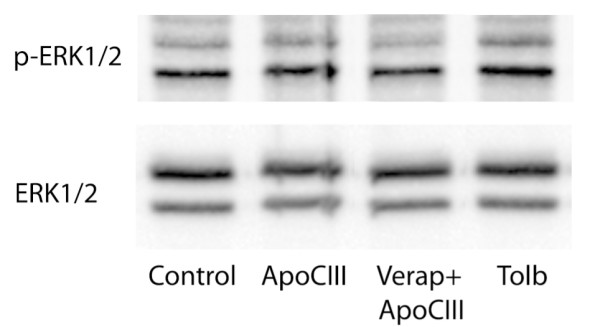
**ApoCIII-induced ERK1/2 activation**. Levels of phosphorylated ERK1/2 in INS-1E cells treated or not with apoCIII were measured after 0.5 hours by immunoblotting. Cells were exposed to L-type Ca^2+ ^channel blocker verapamil (Verap) or K_ATP _channel blocker tolbutamide (Tolb) as indicated.

The role of Ca^2+ ^influx for ERK1/2 activation was next investigated. INS-1E cells treated with apoCIII were exposed to L-type Ca^2+ ^channel blocker verapamil prior to apoCIII-treatment. ApoCIII-induced ERK1/2 activation observed after 0.5 hour was reversed by treatment with the channel antagonist (Fig 3). Further support of a role of Ca^2+ ^influx as activator of ERK1/2 was provided when INS-1E cells were exposed to tolbutamide. After treatment with the K_ATP _channel blocker, elevated levels (p < 0.05) of p-ERK1/2 were observed (Fig 3).

### ApoCIII-induced apoptosis and CHOP

**Figure 4:**
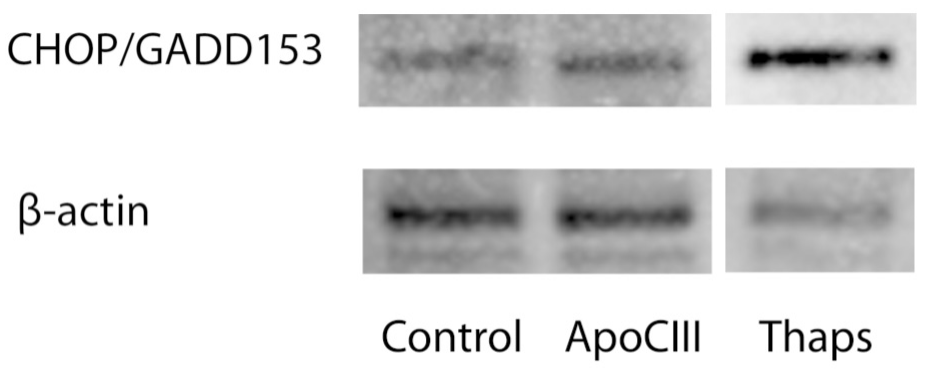
**ApoCIII and CHOP/GADD153**. Levels of CHOP/GADD153 in INS-1E cells treated or not with apoCIII were measured after 24 hours by immunoblotting. Cells treated with 300 nM thapsigargin (Thaps) were used as positive control.

Lastly, we investigated if apoCIII-induced elevated apoptosis in INS-1E cells (Fig 1) involved enhanced expression of pro-apoptotic protein CHOP/GADD153. In the apoCIII-exposed cells CHOP/GADD153 protein levels were not different from those observed under control conditions, however (Fig 4).

## Discussion

The present study indicates that elevated levels of the apolipoprotein apoCIII could promote apoptosis in insulin-producing β-cells via activation of MAPK p38 and ERK1/2. The study is a continuation of our previous study in which apoCIII was identified as an up-regulated protein by proteomic methodology when serum obtained from individuals with newly diagnosed T2DM and documented impaired β-cell function was compared to serum from control individuals with normal β-cell function [9].

ApoCIII is an 8.8 kDa polypeptide synthesized by the liver and playing an important role in controlling catabolism of triglyceride-rich lipoproteins by inhibiting the activity of lipoprotein lipase thereby inducing hypertriglyceridemia [21,22]. The apolipoprotein is mainly associated with HDL but also with LDL and VLDL [23,24]. In individuals with T2DM the levels of the apolipoprotein is elevated [9,13,14]. To what extent the elevated apoCIII levels in T2DM are accounted for by rise in a particular lipoprotein particle class is less clear, however. Although the levels of VLDL in T2DM individuals are increased substantially compared to non-diabetic subjects, apoCIII was not increasing in concert with the increased VLDL concentration and core lipids in these individuals [14]. Instead increased apoCIII content was observed in small dense LDL (sdLDL) in T2DM patients compared to the corresponding fraction from healthy individuals [25]. The potential role of the sdLDL, which is a lipoprotein subclass, was further emphasized by the strong association with coronary disease progression. Based on these results sdLDL and apoCIII have been suggested as markers of the atherogenic dyslipidemia of insulin resistance and type 2 diabetes [25-27]. In addition, connection between genetic alterations in apoCIII and T2DM was recently evidenced by the finding of strong association between changes in *ApoC3 *with lipid derangements in individuals with the disease [28]. When over-expressing apoCIII in transgenic mice, hypertriglyceridemia follows [29]. Conversely, disruption of *ApoC3 *in mice reduced triglyceride levels [30]. Despite the strong correlation relationship between apoCIII and triglyceride levels [13], very little is known about direct effects of the apolipoprotein on the insulin-producing cell.

Elevated apoCIII levels have in one previous study been associated with enhanced apoptosis in insulin-secreting β-cells possibly explained by elevated cytoplasmic Ca^2+ ^levels [15]. Since disturbed Ca^2+ ^homeostasis of the endoplasmic reticulum (ER) would be manifested as elevated cytoplasmic Ca^2+ ^levels, we examined if apoCIII-induced apoptosis was connected to up-regulation of the pro-apoptotic protein CHOP/GADD153. The protein is a down-stream target of the protein kinase R-like ER kinase (PERK) signaling pathway of the ER-stress response [31]. The pathway is part of an adaptive response, the unfolded protein response (UPR), aiming at restoring ER function when mis- or unfolded proteins accumulate in the organelle as a consequence of disturbances in ER Ca^2+^. When the UPR fails, persisting ER-stress follows and apoptosis is elevated, where up-regulation of CHOP/GADD153 is a component part [7,19]. The enhanced apoptosis induced by apoCIII in INS-1E cells in the present study was not associated with enhanced CHOP/GADD153 levels, however.

We also investigated to what extent apoCIII activated MAPKs p38, JNK and ERK1/2. Treating INS-1E cells with apoCIII almost doubled the amounts of p-p38 after 24 hours exposure to the apolipoprotein. A role of this activation in apoCIII-induced apoptosis was supported by the abrogation of apoptosis when inhibitor of the kinase was administered. In addition, activation of the MAPK has been observed in β-cells exposed to elevated levels of fatty acids, oxygen radical formation and cytokine, all conditions connected with enhanced β-cell apoptosis [16,17]. ERK1/2 activation was also induced by apoCIII but with a more rapid and transient phosphorylation pattern. Our observations that administration of the L-type Ca^2+ ^channel antagonist verapamil abrogated rise in p-ERK1/2 induced by apoCIII and that K_ATP _channel blocker tolbutamide induced ERK1/2 activation are supporting a role of Ca^2+ ^influx, induced by apoCIII, as a component of the ERK1/2 activation [15,32]. ERK1/2 activation does not seem to be essential for the short-term performance of the β-cell since glucose-stimulated insulin secretion (GSIS) was not affected by presence of an inhibitor of the kinases [32]. In contrast, when the inhibitor was administered to islets exposed to prolonged elevated glucose levels, which in the absence of the inhibitor caused similar rapid and transient ERK1/2 activation and was associated with impaired glucose-stimulated insulin secretion and apoptosis, improved GSIS and reduced apoptosis was observed [18]. The same beneficial effects were obtained by L-type Ca^2+ ^channel antagonist nimodipine. Thus, apoCIII elevates cytoplasmic Ca^2+ ^levels by promoting Ca^2+ ^influx. Although such influx promotes insulin secretion in the short perspective, prolonged elevated Ca^2+ ^levels are associated with enhanced β-cell apoptosis [15,33]. Among the pro-apoptotic signaling pathways ERK1/2 and p38 activation seem to play fundamental roles since their inhibitions, without Ca^2+ ^antagonism, independently normalized apoCIII-induced apoptosis. In contrast, JNK was not activated by apoCIII.

## Conclusion

In conclusion, the study indicates that elevated levels of the apolipoprotein apoCIII may affect β-cell function via activation of MAPKs p38 and ERK1/2. In addition, negative effects of apoCIII on β-cell function may also be mediated by the rise in circulating triglycerides associated with elevated levels of the apolipoprotein [13,22,34]. In this perspective, therapies aiming at normalizing levels of apoCIII including benzafibrate could prove to be important not only for cardiovascular protection [26] but also to preserve β-cell function.

## Competing interests

The authors declare that they have no competing interests.

## Authors' contributions

EMS was responsible for design, planning, carrying out western blotting of the different MAPK and its linkage to apoCIII-induced apoptosis, calcium dependent ERK1/2 activation, statistical analysis and contributed to write the manuscript. TS carried out the initial experiments of apoptosis-measurements and western blotting of CHOP/GADD153. PB conceived the study, participated in its design and was responsible for writing the manuscript. All authors read and approved the final manuscript.
